# Identification of a prostaglandin D_2_ metabolite as a neuritogenesis enhancer targeting the TRPV1 ion channel

**DOI:** 10.1038/srep21261

**Published:** 2016-02-16

**Authors:** Takahiro Shibata, Katsuhiro Takahashi, Yui Matsubara, Emi Inuzuka, Fumie Nakashima, Nobuaki Takahashi, Daisuke Kozai, Yasuo Mori, Koji Uchida

**Affiliations:** 1 Graduate School of Bioagricultural Sciences, Nagoya University, Nagoya 464-8601, Japan; 2 PRESTO, Japan Science and Technology Agency (JST), Kawaguchi, Saitama 332-0012, Japan; 3 Laboratory of Molecular Biology, Department of Synthetic Chemistry and Biological Chemistry, Graduate School of Engineering, Kyoto University, Kyoto 615-8510, Japan

## Abstract

Mast cells play important roles in allergic inflammation by secreting various mediators. In the present study, based on the finding that the medium conditioned by activated RBL-2H3 mast cells enhanced the nerve growth factor (NGF)-induced neuritogenesis of PC12 cells, we attempted to isolate an active compound from the mast cell conditioned culture medium. Our experiment identified 15-deoxy-Δ^12,14^-PGJ_2_ (15d-PGJ_2_), one of the PGD_2_ metabolites, as a potential enhancer of neuritogenesis. 15d-PGJ_2_ strongly enhanced the neuritogenesis elicited by a low-concentration of NGF that alone was insufficient to induce the neuronal differentiation. This 15d-PGJ_2_ effect was exerted in a Ca^2+^-dependent manner, but independently of the NGF receptor TrkA. Importantly, 15d-PGJ_2_ activated the transient receptor potential vanilloid-type 1 (TRPV1), a non-selective cation channel, leading to the Ca^2+^ influx. In addition, we observed that (i) NGF promoted the insertion of TRPV1 into the cell surface membrane and (ii) 15d-PGJ_2_ covalently bound to TRPV1. These findings suggest that the NGF/15d-PGJ_2_-induced neuritogenesis may be regulated by two sets of mechanisms, one for the translocation of TRPV1 into the cell surface by NGF and one for the activation of TRPV1 by 15d-PGJ_2_. Thus, there is most likely a link between allergic inflammation and activation of the neuronal differentiation.

Mast cells are some of the principal effector cells involved in the pathogenesis of allergic diseases and in certain host responses against infection. The roles of mast cells in mediating an allergic reaction are well understood. The aggregation of the high affinity IgE receptor (FcεRI) on mast cells by allergen/antigen results in the rapid release of pro-inflammatory mediators. This is followed by a delayed phase of mast cell activation resulting in the production and secretion of cytokines and chemokines that recruit other immune cells, such as lymphocytes and eosinophils, leading to chronic inflammation^1^. Although the view that mast cells are primarily involved in the immune responses has been prevalent^2^, there is increasing evidence that mast cells participate in other physiological processes such as nervous system functions^3^.

The activation of mast cells leads to the rapid production of a large variety of mediators, such as prostaglandins (PGs)^4^,^5^. PGD_2_ is known to be one of the major PGs produced from mast cells, which has significant effects on a number of biological processes, such as platelet aggregation, relaxation of smooth muscles, and nerve cell functions. PGD_2_ also exerts its allergic inflammatory effects, including blood flow changes, influx of Th2 lymphocytes and eosinophils, and induction of Th2 cytokine production, through high affinity interactions with the G-protein coupled receptors DP1 and chemoattractant-homologous receptor expressed on T-helper type 2 cells (CRTH2). Both receptors act in concert to facilitate a variety of biological functions involved in the development and maintenance of the allergic response^6^,^7^. Meanwhile, PGD_2_ is a relatively unstable molecule. PGD_2_ readily undergoes spontaneous dehydration in aqueous media to yield biologically active cyclopentenone PGs of the J_2_ series, such as PGJ_2_, Δ^12,14^-PGJ_2_, and 15d-PGJ_2_^8^,^9^,^10^. Unlike other classes of prostanoids, the J-series PGs have their own unique spectrum of biological effects, including anti-inflammatory effects^11^,^12^ and an agonistic effect on the peroxisome proliferator-activated receptor γ (PPARγ)^13^,^14^.

Neurotrophins, such as the nerve growth factor (NGF), act as important regulators of the neuronal survival, growth, development, and functional maintenance in the central and peripheral nervous systems. They also prevent neuronal cell damage under various pathophysiological conditions, including ischemia and neuronal diseases. NGF, the first and best characterized member of the neurotrophin family, induces neuronal differentiation and neuritogenesis by binding to TrkA, a receptor tyrosine kinase for NGF. The ligand-bound NGF receptor undergoes autophosphorylation and associates with multiple signaling proteins through the phosphorylated tyrosine moiety. TrkA activates several small G proteins, such as Rap1, Ras, and the Cdc42-Rac1-RhoA family, as well as pathways mediated by the extracellular signal-regulated kinase (ERK), PI3-kinase/Akt and phospholipase C-γ^15^.

In the current study, based on the finding that the conditioned medium from the activated mast cell with IgE/antigen potently promoted the NGF-induced neurite outgrowth, we attempted to isolate an active compound from the medium and identified a PGD_2_ metabolite as a potential neuritogenesis enhancer. In addition, we characterized the cellular events and established a possible mechanism, in which the PGD_2_ metabolite accelerates the NGF-mediated neuritogenesis via activation of a calcium ion channel.

## Results

### Effect of conditioned medium from activated mast cells on NGF-induced neuritogenesis

**Figure 1:**
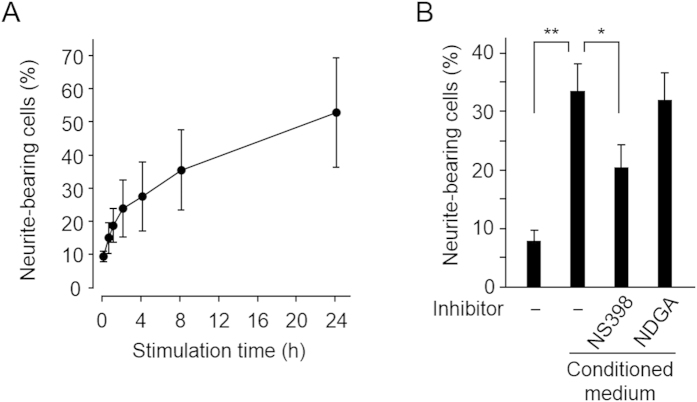
Neuritogenesis-enhancing potency of conditioned medium from mast cells. **(A**) Conditioned medium from stimulated mast cells enhanced the NGF-induced neuritogenesis. The sensitized RBL-2H3 cells were stimulated with 20 ng/ml DNP-BSA for the indicated times (0-24h). The resulting medium was used for the neuritogenesis assay in the PC12 cells treated with 1.5 ng/ml NGF. The results shown are the means ± SD of three independent experiments. (**B**) Effects of COX and LOX inhibitors on neuritogenic potency of the conditioned medium. The sensitized RBL-2H3 cells were pre-treated with NS393 (50 μM) or nordihydroguaiaretic acid (NDGA) (25 μM) for 30 min and then stimulated with 20 ng/ml DNP-BSA for 8h. The resulting conditioned medium was used for the neuritogenesis assay. The results shown are the means ± SD of three independent experiments. **p<0.01, *p<0.05.

Upon activation, mast cells release numerous vasoactive and pro-inflammatory mediators and play an important role in allergic inflammation. To find an active molecule providing a link between inflammation and the neuronal response, we examined the effect of the conditioned medium from the activated mast cells on the NGF-induced neuritogenesis. The RBL-2H3 mast cells were stimulated with anti-DNP IgE/DNP-BSA, and the medium of the activated mast cells was examined for the NGF-induced neuritogenesis of rat pheochromocytoma PC12 cells. As shown in Fig. 1A, the medium significantly enhanced the NGF-induced neurite outgrowth in a time-dependent manner. The pretreatment of the cyclooxygenase (COX) inhibitor NS398 decreased the activity of the medium, whereas the lipoxygenase (LOX) inhibitor, nordihydroguaiaretic acid (NDGA) had little effect (Fig. 1B). These data suggest the involvement of the COX-derived product(s) in the promotion of neuritogenesis.

### Identification of a neuritogenic enhancer in conditioned medium from activated mast cells

**Figure 2:**
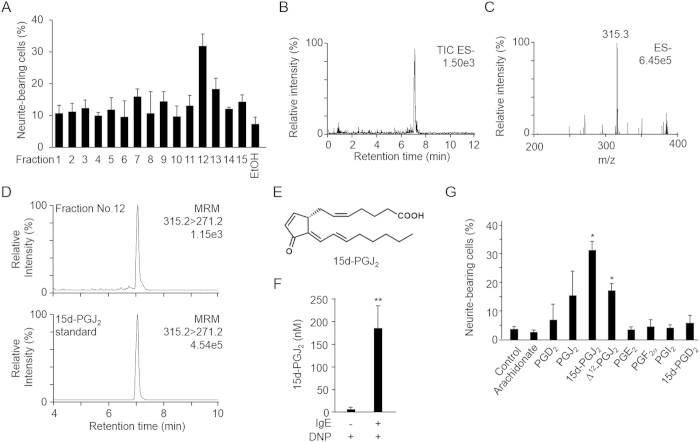
Identification of a neuritogenic enhancer from the conditioned medium of the stimulated-mast cells. (**A)**, Neuritogenesis-promoting potency of fractions separated by reverse-phase HPLC. The ethyl acetate extract of the conditioned medium was fractionated every 2 min and divided into 15 factions by HPLC. The PC12 cells were treated with each fraction and 1.5 ng/ml NGF for 72 h. The results shown are the means ± SD of three independent experiments. B, and C, LC-ESI-MS analysis of the fraction No. 12 in negative ion mode. (**B**) The total ion chromatography. Scanned from m/z 200 to 400. (**C**) Mass spectrum of the peak observed in panel (**B,D**) The ion current tracing of 15d-PGJ_2_ using LC-ESI-MS/MS with MRM mode. *Upper*, fraction No.12; *Lower*, authentic standard. E, Chemical structures of 15d-PGJ_2_. F, Quantification of 15d-PGJ_2_ in the conditioned medium. The results shown are the means ± SD of three independent experiments. **p<0.01. G, Effect of PGs on neuritogenesis. PC12 cells were treated with 1 μM PGs and 1.5 ng/ml NGF for 72 h. The results shown are the means ± SD of three independent experiments. *p<0.05 vs. NGF alone (Control).

To identify the COX products responsible for the synergistic effect on the NGF-dependent neuritogenesis, we conducted an activity-guided separation of a principal enhancer in the conditioned medium from the activated RBL-2H3 cells. The ethyl acetate extract was fractionated into 15 fractions by HPLC using a reverse-phase column, and the resulting fractions were assayed for synergistic activity in the PC12 cells. As shown in Fig. 2A, fraction No. 12 exhibited the most potent enhancing activity. The LC-ESI-MS analysis of fraction No. 12 showed a single peak with a pseudomolecular ion peak at m/z 315.3 (M-H)^−^ (Fig. 2B,C). Among the COX products, a PGD_2_ metabolite, 15-deoxy-Δ^12^,^14^-PGJ_2_ (15d-PGJ_2_; molecular weight 316.4), has a similar molecular mass. Indeed, the main product in fraction No. 12 showed the same retention time as the peak for the authentic 15d-PGJ_2_ (Fig. 2D,E). In addition, when the formation of 15d-PGJ_2_ was examined in the conditioned medium from the activated RBL-2H3 mast cells using the LC-ESI-MS/MS technique (Fig. S1), the stimulation of the cells with the antigen for 8 h gave about 180 nM of 15d-PGJ_2_ in the medium (Fig. 2F). In addition to 15d-PGJ_2_, other PGs, such as PGE_2_, PGD_2_, 15-deoxy-PGD_2_, PGJ_2_, and Δ^12^-PGJ_2_, were also detected in the conditioned medium from the activated mast cells by LC-ESI-MS/MS (Fig. S2). Hence, to further confirm that 15d-PGJ_2_ is the major active component, we examined the effect of the PGs on the NGF-induced neurite outgrowth and found that the neuritogenesis was very potently enhanced by 15d-PGJ_2_ (Fig. 2G and S3). Beside 15d-PGJ_2_, PGJ_2_ and Δ^12^-PGJ_2_ moderately enhanced the neurite outgrowth, suggesting that the J_2_-type PGs could typically act as a neuritogenic enhancer. Indeed, J_2_-type PGs including 15d-PGJ_2_ were previously reported to promote the NGF-induced neurite outgrowth in PC12h cells by Satoh *et al*.^16^.

### 15d-PGJ_2_ accelerates neuritogenesis in cooperation with NGF

**Figure 3:**
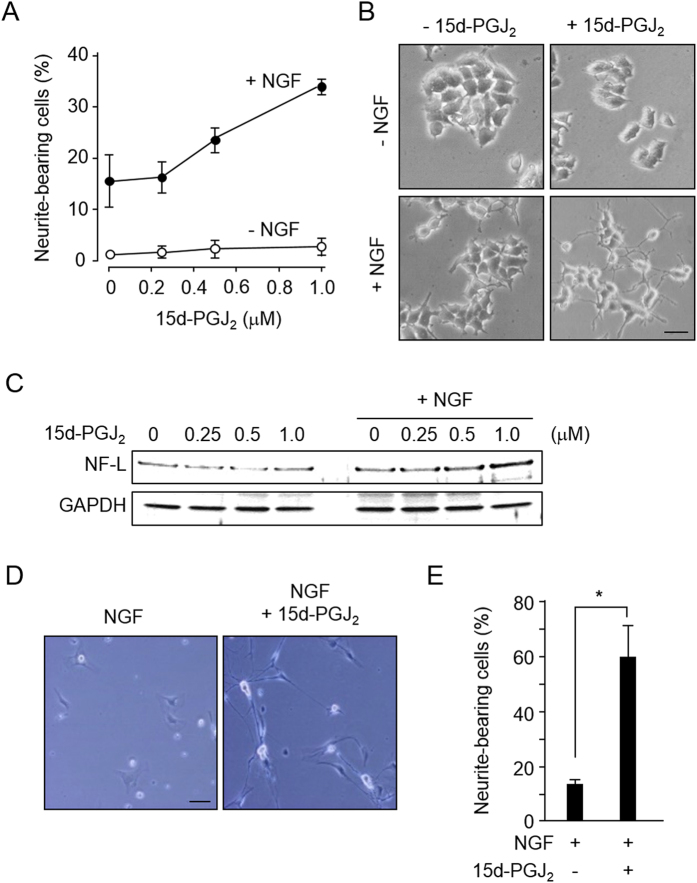
15d-PGJ2 enhances NGF-induced neurite outgrowth. (**A**), Dose-dependent enhancement of NGF-induced neurite outgrowth. The PC12 cells were treated with 15d-PGJ_2_ (0-1 μM) in the absence (*open circle*) or presence (*closed circle*) of 1.5 ng/ml NGF for 72 h. The results shown are the means ±SD of three independent experiments. (**B**) Representative images of PC12 cells treated with 1 μM 15d-PGJ_2_ in the absence or presence of NGF (1.5 ng/ml) for 72 h. Scale bars, 10 μM. (**C**) Dose-dependent induction of the NF-L protein in PC12 cells. The cells were treated with 15d-PGJ_2_ in the presence or absence of 1.5 ng/ml NGF for 72 h. (**D**) and E, 15d-PGJ_2_ enhanced neuritogenesis in DRG cells. The DRG cells were treated with 1 μM 15d-PGJ_2_ in the presence of NGF (1.5 ng/ml) for 72 h. D, representative images. Scale bars, 10 μM. (**E**) The results shown are the means ± SD of three independent experiments. *p < 0.05 vs. NGF alone.

As shown in Fig. 3A,B, 15d-PGJ_2_ had no significant effect on the neurite outgrowth in the absence of NGF, whereas it strongly enhanced the neuritogenesis elicited by a low-concentration of NGF that alone was insufficient to induce neuronal outgrowth. Consistent with these results, 15d-PGJ_2_ alone had little effect on the expression of a neurofilament light (NF-L) protein, a neuronal differentiation marker, whereas the combined stimulus of 15d-PGJ_2_ and NGF enhanced the expression of NF-L (Fig. 3C). The 15d-PGJ_2_-enhanced neurite outgrowth was also observed in the rat primary dorsal root ganglia (DRG) cells and the level of outgrowth in the DRG cells exposed to 15d-PGJ_2_/NGF was ∼4-fold higher than that in the cells exposed to NGF alone (Fig. 3D,E). Thus, 15d-PGJ_2_ accelerates the neuritogenesis in cooperation with NGF.

### 15d-PGJ_2_ accelerates NGF-induced neuritogenesis in a Ca^2+^-dependent manner

**Figure 4:**
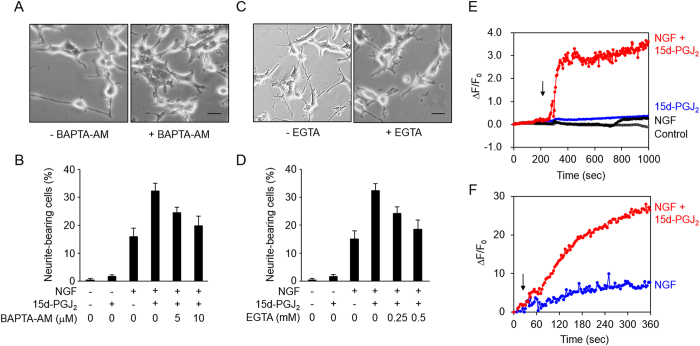
Involvement of intracellular Ca2+ in 15d-PGJ2-enhanced neuritogenesis. (**A,B**) Effect of a membrane-permeable Ca^2+^ chelator BAPTA-AM on 15d-PGJ_2_-enhanced neuritogenesis. The PC12 cells were pre-treated with BAPTA-AM for 30 min, then stimulated with 1 μM 15d-PGJ_2_ in the presence of 1.5 ng/ml NGF for 72 h. A, Representative images. Scale bars, 10 μM. B, The results shown are the means ± SD of three independent experiments. (**C,D**) Effect of a membrane-impermeable Ca^2+^ chelator, EGTA, on 15d-PGJ_2_-enhanced neuritogenesis. The PC12 cells were pre-treated with EGTA for 30 min, then stimulated with 1 μM 15d-PGJ_2_ in the presence of 1.5 ng/ml NGF for 72 h. C, Representative images. Scale bars, 10 μM. D, The results shown are the means ± SD of three independent experiments. (**E,F**) Ca^2+^ increases induced by 15d-PGJ_2_ (1 μM) with or without NGF (1.5 ng/ml) in the PC12 cells (**E**) and DRG cells (**F**). The arrow indicates the time of treatment.

It has been established that NGF induces cell differentiation and neurite outgrowth via the TrkA receptor^17^,^18^. However, 15d-PGJ_2_ did not have any effect on the NGF-induced autophosphorylation of TrkA (Fig. S4A), suggesting that 15d-PGJ_2_ might act downstream of the NGF-TrkA signaling pathway. Indeed, downstream signaling pathways, including ERK, JNK, and PKC, were enhanced by the co-treatment of 15d-PGJ_2_ and NGF (Fig. S4B–D). To gain insight into the molecular mechanism underlying the acceleration of the NGF-induced neurite outgrowth by 15d-PGJ_2_, we tested several small molecule inhibitors directed against the downstream signaling molecules of the NGF-TrkA pathway and found that the neuritogenesis was most effectively inhibited by the Ca^2+^ chelators. The pretreatment of the 1,2-bis-(2-amino-phenoxy)ethane-*N,N,N’,N’*-tetraacetic acid acetoxymethyl ester (BAPTA-AM), a cell-permeable Ca^2+^ chelator, significantly inhibited the 15d-PGJ_2_-enhanced neurite outgrowth (Fig. 4A,B). Similar results were also observed when the cells were treated with EGTA, an extracellular calcium chelator (Fig. 4C,D). In addition, the Ca^2+^ ionophore ionomycin dramatically enhanced the neuritogenesis in PC12 cells treated with NGF (Fig. S5). In addition, we measured the changes in the intracellular Ca^2+^ levels in PC12 cells using the Ca^2+^-specific fluorescence probe, Fluo-8, and found that the co-treatment of 15d-PGJ_2_ and NGF significantly increased the Ca^2+^ levels in the PC12 (Fig. 4E) and DRG cells (Fig. 4F). Thus, it appeared that 15d-PGJ_2_ acts on a Ca^2+^-dependent signaling pathway.

### Involvement of TRPV1 in the 15d-PGJ_2_/NGF-induced neuritogenesis

**Figure 5:**
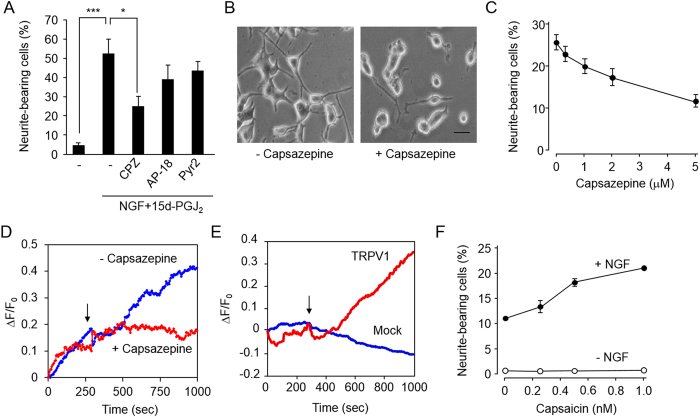
Involvement of TRPV1-dependent Ca2+ influx on 15d-PGJ2-enhanced neuritogenesis. (**A**,**B**) Effect of TRP channel inhibitors on 15d-PGJ_2_-enhanced neuritogenesis. The PC12 cells were pre-treated with TRPV1 inhibitor, Capsazepine (CPZ) (5 μM), TRPA1 inhibitor AP-18 (10 μM), or TRPC inhibitor Pyr2 (0.1 μM) for 30 min, then stimulated with 1 μM 15d-PGJ_2_ in the presence of 1.5 ng/ml NGF for 72 h. The results shown are the means ± SD of three independent experiments (Panel A). ***p<0.005, *p<0.05. B, Representative images. Scale bars, 10 μM. (**C**), Dose-dependent inhibitory effect of Capsazepine on 15d-PGJ_2_-enhanced neuritogenesis. The results shown are the means ± SD of three independent experiments. (**D**) Effect of Capsazepine (5 μM) on the Ca^2+^ increases induced by 15d-PGJ_2_ (1 μM) in the PC12 cells. The arrow indicates the time of treatment. (**E**) Ca^2+^ increases induced by 15d-PGJ_2_ (1 μM) in HEK293 cells transfected with expression plasmids for empty vector (Mock) or TRPV1. The arrow indicates the time of treatment. (**F**) Effect of Capsaicin on neuritogenesis. The PC12 cells were treated with the indicated concentrations of Capsaicin in the absence (*open circle*) or presence (*closed circle*) of 1.5 ng/ml NGF for 72 h. The results shown are the means ±SD of three independent experiments.

Based on the previous findings that 15d-PGJ_2_ could target the Ca^2+^ channels, such as TRP ankyrin-1 (TRPA1)^19^,^20^,^21^,^22^, we tested the effect of the Ca^2+^ channel inhibitors on the 15d-PGJ_2_/NGF-induced neuritogenesis. Capsazepine, a TRP vanilloid receptor 1 (TRPV1) inhibitor, showed the most significant inhibitory effect (Fig. 5A–C). However, the inhibitors directed against TRPA1 (AP-18) and TRPC (Pyr2) were only slightly inhibitory (Fig. 5A). Other inhibitors, including the L-type Ca^2+^ channel and Na^+^ channel inhibitors, did not show any effect (Fig. S6). In addition, the TRPV1 channel inhibitor inhibited the 15d-PGJ_2_-induced increase in intracellular Ca^2+^ level in the PC12 cells (Fig. 5D). We also observed that (i) 15d-PGJ_2_ increased the intracellular Ca^2+^ level in the HEK293 cells transfected with TRPV1 cDNA (Fig. 5E), (ii) the effect of 15d-PGJ_2_ on the NGF-induced neuritogenesis was fully reproduced by treatment of the cells with capsaicin, a TRPV1 agonist (Fig. 5F), (iii) capsaicin-enhanced neuritogenesis was inhibited by the treatment of BAPTA-AM (Fig. S7A,B), and (iv) the TRPV1 channel inhibitor significantly reduced the neuritogenesis in the DRG cells (Fig. S7C). These data strongly suggest the involvement of TRPV1 in the 15d-PGJ_2_-enhanced neuritogenesis.

**Figure 6:**
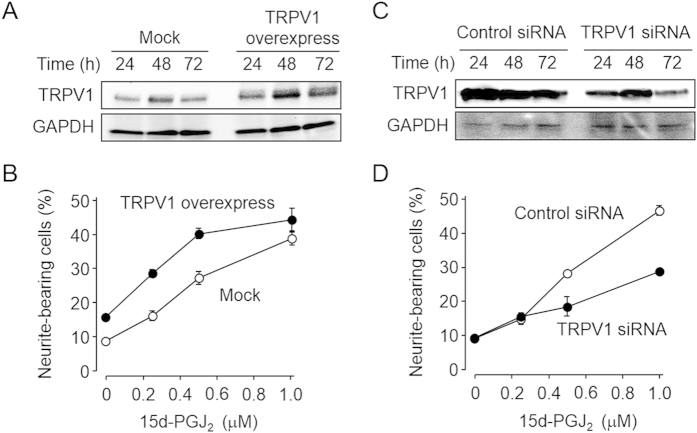
TRPV1-dependent neuritogenesis induced by 15d-PGJ2. (A,B) Effect of TRPV1 overexpression on neuritogenesis. The transfected cells were stimulated with 15d-PGJ_2_ in the presence of 1.5 ng/ml NGF for 72 h. The results shown are the means ± SD of three independent experiments. Open circle, Empty vector (Mock); closed circle, expression plasmid for TRPV1. **(C,D**) Effect of the control siRNA (*open circle*) and the specific siRNA of the TRPV1 (*closed circle*) on neuritogenesis. The transfected cells were stimulated with 15d-PGJ_2_ in the presence of 1.5 ng/ml NGF for 72 h. The results shown are the means ± SD of three independent experiments.

To further confirm the involvement of TRPV1 in the 15d-PGJ_2_/NGF-induced neuritogenesis, we examined the effect of the TRPV1 overexpression on the neuritogenesis. PC12 cells were transfected with the expression plasmids for the TRPV1 or empty vector and were stimulated with 15d-PGJ_2_ together with NGF. The expression level of TRPV1 was assessed by immunoblotting with the anti-TRPV1 antibody (Fig. 6A). As shown in Fig. 6B, neurite outgrowth was significantly enhanced by the transfection of TRPV1 (Fig. 6B). In addition, we also examined the effect of the TRPV1 down-regulation on the 15d-PGJ_2_-enhanced neuritogenesis using RNAi and observed that the siRNA of the TRPV1 resulted in the corresponding changes in the expression of TRPV1 (Fig. 6C). The knock down of TRPV1 reduced the neurite outgrowth enhanced by 15d-PGJ_2_ (Fig. 6D). These data also support our hypothesis and confirmed that 15d-PGJ_2_ acts as an enhancer of neuritogenesis via activation of TRPV1.

### 15d-PGJ_2_ as a covalent ligand of TRPV1

**Figure 7:**
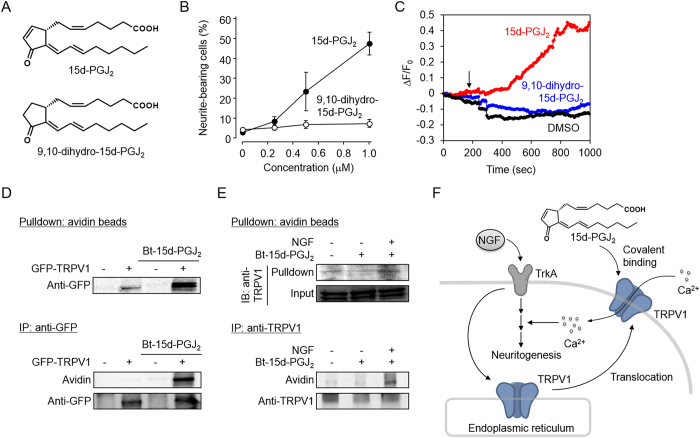
Covalent modification of TRPV1 by 15d-PGJ2 in living cells. **(A**) Chemical structures of 15d-PGJ_2_ (*upper*) and 9,10-dihydro-15d-PGJ_2_ (*lower*). (**B**) Effect of 9,10-dihydro-15d-PGJ_2_ on NGF-dependent neuritogenesis. The PC12 cells were treated with 15d-PGJ_2_ (*closed circle*) or 9,10-dihydro-15d-PGJ_2_ (*open circle*) in the presence of 1.5 ng/ml NGF for 72 h. The results shown are the means ± SD of three independent experiments. (**C**) Effect of 9,10-dihydro-15d-PGJ_2_ (1 μM) on Ca^2+^ increase in the PC12 cells. The arrow indicates the time of treatment. (**D**) Interaction between TRPV1 and 15d-PGJ_2_ in TRPV1-expressing HEK293 cells. HEK293 cells transfected with GFP-tagged TRPV1 were treated with 1 μM biotinylated 15d-PGJ_2_ (Bt-15d-PGJ_2_) for 10 min. Cell lysates were incubated with immobilized NeutrAvidin or with anti-GFP-beads, as indicated. (**E**) Interaction between TRPV1 and 15d-PGJ_2_ in the intact PC12 cells. The PC12 cells were treated with Bt-15d-PGJ_2_ (1 μM) together with or without 1.5 ng/ml NGF for 10 min. The cell lysates were incubated with immobilized NeutrAvidin or with anti-TRPV1-beads, as indicated. (**F**) Model for mechanisms by which 15d-PGJ_2_ enhances the NGF-induced neuritogenesis via TRPV1 activation.

It has been established that the electrophilic α,β-unsaturated carbonyl group in the cyclopentenone ring of 15d-PGJ_2_ is essential for a variety of biological functions^23^. Hence, we tested the effect of 9,10-dihydro-15d-PGJ_2_, lacking the electrophilic α,β-unsaturated functionality (Fig. 7A), on the NGF-induced neuritogenesis. As shown in Fig. 7B, the NGF-induced neurite outgrowth was enhanced by 15d-PGJ_2_ in a dose-dependent manner. In contrast, 9,10-dihydro-15d-PGJ_2_ had no significant effects on the neuritogenesis. In a manner similar to the induction of the neuritogenesis, 15d-PGJ_2_ resulted in a rapid increase in the intracellular Ca^2+^ level, whereas the Ca^2+^ levels were maintained at about the basal level in the cells exposed to 9,10-dihydro-15d-PGJ_2_ (Fig. 7C). Thus, the reduction of the double bond in the cyclopentenone ring virtually abolished the neuritogenic activity and induction of the Ca^2+^ influx of 15d-PGJ_2_, indicating that the neuritogenesis-enhancer activity can be attributed to the electrophilic center of 15d-PGJ_2_.

Finally, to examine whether 15d-PGJ_2_ could covalently bind TRPV1 in the intact cells, we incubated the GFP-tagged TRPV1-introduced HEK293 cells with a biotinylated 15d-PGJ_2_ (Bt-15d-PGJ_2_), which retains the α,β-unsaturated ketone substituent and the neuritogenesis-enhancing activity (Fig. S8), followed by Avidin pull-down and immunoprecipitation experiments. The cell lysates were incubated with NeutrAvidin beads, and the biotinylated proteins bound to the beads were then eluted and analyzed by immunoblot using the anti-GFP antibody. As shown in Fig. 7D, the GFP-tagged TRPV1 was detected in the precipitate from the Bt-15d-PGJ_2_-treated cells. Alternatively, the cell lysates were subjected to immunoprecipitation with an anti-GFP antibody, and the presence of the Bt-15d-PGJ_2_-modified proteins was detected using HRP-conjugated NeutrAvidin (Fig. 7D). In addition, we investigated the covalent modification of endogenous TRPV1 in intact PC12 cells in the presence or absence of NGF. Intriguingly, pull-down and immunoprecipitation studies showed that 15d-PGJ_2_ could react with TRPV1 only in the presence of NGF in the PC12 cells (Fig. 7E). More strikingly, consistent with the observations that NGF induced the translocation of TRPV1 into the cell membrane (Fig. S9A), immunocytochemical studies showed colocalization of Bt-15d-PGJ_2_ and TRPV1 in the cell surface membrane of the NGF-treated PC12 cells (Fig. S10).These and the findings that (i) specific inhibitor for PI3K, which regulates the translocation and sensitization of TRPV1^24^,^25^, inhibited the 15d-PGJ_2_-enhanced neuritogenesis without showing cytotoxicity (Fig. S9B,C), (ii) 15d-PGJ_2_-enhanced PKC activation was reduced by the treatment of PI3K inhibitor and TRPV1 antagonist (Fig. S11), suggest the involvement of NGF-induced translocation of TRPV1 through PI3K signaling in the 15d-PGJ_2_-enhanced neuritogenesis.

## Discussion

In the present study, based on the finding that the conditioned medium from the activated RBL-2H3 mast cells accelerated the NGF-induced neuritogenesis of the PC12 cells, we attempted to separate and purify the active compound from the conditioned medium and identified 15d-PGJ_2_, one of the PGD_2_ metabolites, as an enhancer of the NGF-induced neuritogenesis. Moreover, using LC-ESI-MS/MS in the MRM mode, we extensively analyzed PGD_2_ and its metabolites in the medium of stimulated mast cells and detected 15d-PGJ_2_ as a major product. Strikingly, the maximum yield of 15d-PGJ_2_ was about 180 nM. The amounts of 15d-PGJ_2_ generated, at least *in vitro*, may be sufficient for it to play a role in modulating most of the responses. These data suggested that 15d-PGJ_2_ might represent a *bona fide* active component essential for the induction of neuritogenesis by NGF and that the neuronal response during mast cell degranulation might be ascribed, at least in part, to the production of PGD_2_ followed by the conversion to 15d-PGJ_2_.

It has been previously established^8^,^9^,^10^ that PGD_2_ can be spontaneously converted to 15d-PGJ_2_ as follows: (i) PGD_2_ is initially converted to the dehydration products, PGJ_2_; (ii) PGJ_2_ is isomerized to an α,β-unsaturated enone intermediate followed by dehydration to form 15d-PGJ_2_. Although it remains unknown if this PGD_2_ metabolism is utilized in an organism, 15d-PGJ_2_ has been detected in many types of human and animal samples. The presence of 15d-PGJ_2_ *in vivo* was first demonstrated in the inflammatory exudates of the carrageenin-induced pleurisy^26^. Later, using a murine monoclonal antibody against 15d-PGJ_2_, the accumulation of 15d-PGJ_2_ was demonstrated in the cytoplasm of most of the foamy or spindle macrophages in human atherosclerotic plaques^10^ and in the spinal cord of sporadic amyotrophic lateral sclerosis (ALS) patients^27^. These findings suggested that, because PGD_2_ is the major prostanoid in most tissues, 15d-PGJ_2_ could be produced at a number of sites and ubiquitously involved in inflammation and its related disorders.

Our current study showed that 15d-PGJ_2_ enhanced the neuritogenesis in an NGF-dependent manner. However, the NGF-induced autophosphorylation of the NGF receptor (TrkA) was not affected by 15d-PGJ_2_ (Fig. S4). This is in striking contrast to the previous finding that an enhanced autophosphorylation of the receptor is involved in the enhancement of the NGF-induced neuritogenesis by an electrophilic isothiocyanate compound^28^. On the other hand, based on the fact that the effect of 15d-PGJ_2_ on the neuritogenesis is NGF-dependent, we speculated that 15d-PGJ_2_ might act somewhere downstream on the NGF-TrkA signaling pathway. A crucial hint for the molecular mechanism was obtained when we tested several inhibitors directed against the downstream signaling molecules of the NGF-TrkA pathway. Among the tested inhibitors, Ca^2+^ chelators significantly inhibited the 15d-PGJ_2_/NGF-induced neuritogenesis (Figs 4 and 5). 15d-PGJ_2_ indeed enhanced the Ca^2+^ influx and, in addition, the Ca^2+^ ionophore dramatically enhanced the NGF-induced neuritogenesis (Fig. S5). Thus, it was anticipated that the Ca^2+^ influx might play a key role in the 15d-PGJ_2_-enhanced neuritogenesis. Indeed, the increase in the intracellular Ca^2+^ concentration via activation of the intracellular sigma-1 and IP3 receptors has been shown to facilitate the NGF-induced neuritogenesis of the PC12 cells^29^.

The involvement of the Ca^2+^ influx in the enhancement of the NGF-induced neuritogenesis by 15d-PGJ_2_ led us to assume that a Ca^2+^ channel might be involved in the mechanisms. Hence, we tested several agonists and antagonists of the Ca^2+^ channels and putatively identified TRPV1 as a key regulator of the NGF/15d-PGJ_2_-induced neuritogenesis. This result was further confirmed by the transfection experiments (Fig. 6). These findings suggest the existence of a mechanism whereby 15d-PGJ_2_ induces the Ca^2+^ influx via activation of a Ca^2+^ channel protein (TRPV1), leading to acceleration of the NGF-TrkA signaling pathway. On the other hand, it was previously reported that CRTH2, a PGD_2_ receptor, is involved in the 15d-PGJ_2_-enhanced neuritogenesis of the PC12 cells^30^. However, PGD_2_, a CRTH2 ligand, was found to be a weak enhancer of the neuritogenesis (Fig. 2G). In addition, the selective CRTH2 agonist, 13,14-dihydro-15-keto-PGD_2_, failed to influence the neuritogenesis regardless of the presence of NGF^30^. Thus, the involvement of CRTH2 as the target of 15d-PGJ_2_ in the NGF-induced neuritogenesis is still questionable.

The TRP superfamily is composed of six subfamilies. TRPV1, among them, is a thermosensitive, nonselective cation channel and can be activated by noxious heat and acidic conditions. It can also recognize a variety of small molecules, such as acrolein and capsaicin, as ligands^31^. We have confirmed that 15d-PGJ_2_ directly interacted with the TRPV1 protein when the GFP-tagged TRPV1-introduced HEK293 cells were incubated with a biotinylated 15d-PGJ_2_ (Bt-15d-PGJ_2_) (Fig. 7). Modification of the cysteine residues in the TRPV1 could be associated with the activation of its channel activity^32^,^33^,^34^,^35^. It has also been reported that 15d-PG_2_ covalently modifies another TRP cannel, TRPA1, to activate the channel protein^19^,^20^,^21^,^22^. Thus, it is not unlikely that the binding of 15d-PG_2_ to TRPV1 might lead to the activation of the TRPV1 channel protein. Although regulation of the 15d-PGJ_2_-mediated TRPV1 activation and its significance need to be further confirmed *in vivo* in future studies, our *in vitro* studies demonstrated that the allergic inflammation, generating PGD_2_ and its metabolites, may result in the neuronal response via activation of the TRPV1.

Zhang *et al*.^24^ have previously shown that NGF activates the PI3K-Src signaling pathway, followed by the phosphorylation and translocation of TRPV1 into the cell membrane. Indeed, we characterized the cellular events triggered by the combined stimulus of NGF and 15d-PGJ_2_ and observed that NGF promoted the insertion of TRPV1 into the cell surface membrane, and the PI3K specific inhibitor LY294002 significantly inhibited the neuritogenesis (Fig. S9). Thus, it is likely that the NGF-induced translocation of TRPV1 into the cell surface may play an essential role in the 15d-PGJ_2_-accelerated neuritogenesis. On the other hand, Ca^2+^ influx through the TRP channels leads to mitogen-activated protein kinase (MAPK) activation in the neuronal cells^36^. In addition, studies using selective pharmacological inhibitors and constitutive-active/dominant-negative mutants have demonstrated the essential role for the MAPKs in the NGF-induced PC12 differentiation^37^,^38^,^39^,^40^. We indeed observed that 15d-PGJ_2_ enhanced the phosphorylation of the MAPKs, such as ERK and JNK, as well as the neuritogenesis (Fig. S4B,C). Thus, the Ca^2+^ influx through the TRPV1 followed by activation of the MAPKs may represent a key mechanism for the 15d-PGJ_2_-enhanced neuritogenesis. Taken together, the NGF/15d-PGJ_2_-induced neuritogenesis may be regulated by two sets of mechanisms, one for the translocation of TRPV1 into the cell surface by NGF and one for the activation of TRPV1 followed by the Ca^2+^ influx by 15d-PGJ_2_ (Fig. 7F).

In summary, we screened PGs based on the neurite outgrowth-inducing effect and identified 15d-PGJ_2_ as a neuritogenic enhancer derived from activated mast cells. Furthermore, we identified the TRPV1 as a molecular target for the neuritogenesis by 15d-PGJ_2_, presenting a molecular basis for the neuronal differentiation by endogenous lipid mediators. Although the endogenous production of 15d-PGJ_2_ needs to be confirmed in future studies, our observation suggest that 15d-PGJ_2_ may act as a mediator that enhances the neuritogenesis. Our current findings may provide clues to the physiological and/or pathophysiological mechanisms of neuritogenesis.

## Methods

### Materials

The antibodies against neurofilament-L and GAPDH were purchased from Chemicon. The anti-GFP-tag and anti-TRPV1 antibodies were from MBL, and Santa Cruz Biotechnology, respectively. The anti-PKCβ antibody was obtained from BD Transduction Laboratories. The antibodies against phospho-ERK, phospho-p38, and phospho-JNK were from Cell Signaling Technology. The recombinant β-NGF was obtained from R&D systems, Inc. PGs, and NS398 were purchased from Cayman Chemical. Anti-flotillin antibody, Nordihydroguaiaretic acid (NDGA), Capsaicin, and Capsazepine, were obtained from Sigma. Preparation of biotinylated 15d-PGJ_2_ was described previously^41^.

### Cell Culture and Neurite Outgrowth Assay

The culture of PC12 cells and neurite outgrowth assay were performed as previously described^28^.

### Preparation of conditioned medium from rat mast cell RBL-2H3

The rat RBL-2H3 mast cell line was obtained from the Human Science Research Resource Bank (Japan). RBL-2H3 cells were grown in DMEM supplemented with 10% FBS, penicillin (100 units/ml), streptomycin (100 μg/ml), and 0.2% NaHCO_3_ at 37 °C in an atmosphere of 95% air and 5% CO_2_. For preparation of the conditioned medium, the RBL-2H3 cells were sensitized overnight with 100 ng/ml anti-DNP IgE (Sigma), then washed and stimulated with 20 ng/ml DNP-BSA (Calbiochem) for the indicated times (0-24h). The resulting medium was used for the neuritogenesis assay and the quantification of 15d-PGJ_2_.

### Isolation of active component from the conditioned medium from activated mast cells

The conditioned medium from the activated RBL-2H3 cells was extracted with ethyl acetate. The resulting ethyl acetate extract was separated by reverse-phase HPLC on a C18 column (Sunniest C18, 4.6 × 250 mM; ChromaNik) at the flow rate of 0.8 ml/min. A gradient was used by solvent A (H_2_O containing 0.1% trifluoroacetic acid) and solvent B (acetonitrile) as follows: time = 0–5 min, 40% B; 35 min, 100% B; 40 min, 100% B. The extract was fractionated in two-minute intervals from 5 to 35 min. The resulting fractions (15 fractions) were evaporated to dryness and redissolved in ethanol.

### LC-ESI-MS/MS analysis for quantification of 15d-PGJ_2_

The conditioned medium was partially separated using Sep-Pak C18 cartridges (Waters). The internal standard, 15d-PGJ_2_-d_4_, was added to the samples prior to the column separation. After the sample loading, the Sep-Pak cartridges were washed with 5  ml of H_2_O containing 0.1% TFA, and the PGs were eluted with 5 ml of ethyl acetate. The samples were then dried, dissolved in ethanol, and subjected to UPLC-ESI-MS analysis. The UPLC-EIS-MS/MS analyses were performed using a Xevo TQD triple quadrupole mass spectrometer system (Waters). The UPLC used a reverse-phase C30 column (Develosil HB C30-UG-3, 100 mM × 2.0 mM, Nomura Chemical, Japan) operated in a column oven set at 40 °C. A discontinuous gradient used solvent A (H_2_O containing 2% isopropanol and 0.05% formic acid) with solvent B (acetonitrile containing 2% isopropanol and 0.05% formic acid) as follows: 40% B at 0 min, 40% B at 1 min, 48% B at 2 min, 48% B at 5 min, 94% B at 6.5 min, 94% B at 12 min, at the flow rate of 0.3ml/min. The negative ion mode MRM transitions were monitored as follows: 15d-PGJ_2_, m/z 315.2 > 271.2 (cone potential 25 eV/collision energy 12 eV); 15d-PGJ_2_-d_4_, m/z 319.2 > 275.2(cone potential 25 eV/collision energy 12 eV); Δ^12^-PGJ_2_, PGJ_2_, and PGJ_2_, m/z 333.2 > 271.2 (cone potential 20 eV/collision energy 14 eV); PGD_2_, and PGE_2_, m/z 351.2 > 271.2 (cone potential 20 eV/collision energy 15 eV). The amount of 15d-PGJ_2_ was quantified by the ratio of the peak area of the target 15d-PGJ_2_ and of the 15d-PGJ_2_-d_4_. QuanLynx software (Waters) was used to create the standard curve and to calculate the 15d-PGJ_2_ concentrations.

### SDS-PAGE and immunoblot analysis

The SDS-PAGE and immunoblot analysis were performed as previously described^28^. Total plasma membrane proteins were extracted using plasma membrane protein extraction kit (101Bio, Palo Alto, CA).

### Isolation of rat DRG neuron

Isolation of the DRG neuron was performed as previously described^18^ with a few modifications. The DRG from the thoracic and lumbar spinal cord of 1–3 day-old rats were cut in small pieces and incubated for 1h at 37 °C in a solution containing 124 mM NaCl, 5 mM KCl, 1.2 mM KH_2_PO_4_, 1.3 mM MgSO_4_, 2.4 mM CaCl_2_, 24 mM NaHCO_3_, 10 mM Glucose, 1.6 mg/ml collagenase type II (Sigma), and 1.6 mg/ml trypsin (Difco Laboratories). They were gently triturated with a fire-polished glass pipette and the resulting solution was centrifuged at 800 rpm for 2 min. The obtained pellet was resuspended in DMEM-F12 HAM medium (Sigma) containing 10% FBS, penicillin and streptomycin. Cells were plated onto dishes coated with poly-L-lysine (Sigma). The cells were cultured in the DMEM-F12 HAM medium containing 10% FBS at 37 °C under 5% CO_2_. All animal protocols were approved by the Animal Experiment Committee in the Graduate School of Bioagricultural Sciences of Nagoya University.

### Ca^2+^ imaging

The cells were seeded in a poly-L-lysine-coated glass bottom dish and cultured for overnight. Assay buffer (140 mM NaCl, 5 mM KCl, 1 mg MgCl_2_, 1 mM Na_2_HPO_4_, 10 mM glucose, 10 mM HEPES, 2 mM CaCl_2,_ and 1.5 mM Probenecid; pH = 7.3) and loading buffer (assay buffer containing 5 μM Fluo-8 AM (ABD Bioquest, Inc.)) were freshly prepared. After washing twice with the assay buffer, the cells were incubated with loading buffer for 30 min, then incubated with the assay buffer for 15 min. Fluorescence measurements were performed using a confocal microscope (FV1000D IX81; Olympus Optical Co., Ltd.).

### Transfection in PC12 Cells

Transfections using Lipofectamine 2000TM (Life technologies) was performed as previously described^28^.

### Pull-down Assay in HEK293 cells

The HEK293 cells transfected with empty or GFP-tagged TRPV1 were treated with 1 μM Bt-15d-PGJ_2_ for 10 min. After treatment, the cells were washed twice with cold PBS and lysed with RIPA buffer containing the protease inhibitor cocktail, and the resulting lysate was treated with NaBH_4_ (20 mM) for 1 h on ice. After removing NaBH_4_ by ultrafiltration, the cell lysate was incubated with immobilized NeutrAvidin or with anti-GFP-Protein G beads, as indicated, at 4 ^o^C overnight. After incubation, the beads were washed three times with lysis buffer, then boiled with the SDS sample buffer. The presence of the GFP-tagged TRPV1 was detected by immunoblotting with the anti-GFP antibody, and the incorporation of Bt-15d-PGJ_2_ into the TRPV1 immunoprecipitates was detected with HRP-NeutrAvidin.

### Pull-down Assay in PC12 cells

The PC12 cells were treated with 1 μM Bt-15d-PGJ_2_ with or without 1.5 ng/ml NGF for 10 min. After treatment, the cells were washed twice with cold PBS and lysed with RIPA buffer containing the protease inhibitor cocktail. The cell lysate was incubated with immobilized NeutrAvidin or with anti-TRPV1-Protein G beads, as indicated, at 4 ^o^C overnight. After incubation, the beads were washed three times with RIPA buffer, then boiled with the SDS sample buffer. The presence of TRPV1 was detected by immunoblotting with the anti-TRPV1 antibody, and the incorporation of Bt-15d-PGJ_2_ into the TRPV1 immunoprecipitates was detected with HRP-NeutrAvidin.

### Immunocytochemical staining

Cells were fixed with 4% paraformaldehyde in PBS for 15 min at room temperature, then permeabilized by treatment with PBS containing 0.5% Triton X-100. The cells were sequentially incubated in 1% bovine serum albumin in PBS for 1h, then incubated with the primary anti-TRPV1 at 4 ^o^C overnight. The cells were then washed with PBS and incubated for 1 h in the presence of Alexa488-labeled donkey anti-goat IgG (Molecular Probes) and Cy5-labeled Avidin (GE Healthcare), rinsed with PBS, and covered with anti-fade solution. The immunofluorescent images were analyzed using a confocal microscope (FV1000; Olympus Optical Co., Ltd., Tokyo, Japan).

## Additional Information

**How to cite this article**: Shibata, T. *et al*. Identification of a prostaglandin D_2_ metabolite as a neuritogenesis enhancer targeting the TRPV1 ion channel. *Sci. Rep.* **6**, 21261; doi: 10.1038/srep21261 (2016).

## Supplementary Material

Supplementary Information
