# Supplementary material for: Identification of a prostaglandin D2 metabolite as a neuritogenesis enhancer targeting the TRPV1 ion channel
Source: Sci Rep. 2016 Feb 16;6:21261. doi: 10.1038/srep21261 (PMC4754695; doi:10.1038/srep21261)
Supplement: Supplementary Information [file srep21261-s1.pdf]

Supplementary Information

Identification of a prostaglandin D_2 metabolite as a neuritogenesis enhancer targeting the TRPV1 ion channel

Takahiro Shibata[a, b], Katsuhiro Takahashi[a], Yui Matsubara[a], Emi Inuzuka[a], Fumie Nakashima[a], Nobuaki Takahashi[c], Daisuke Kozai[c], Yasuo Mori[c], and Koji Uchida[a, *]

[a]Graduate School of Bioagricultural Sciences, Nagoya University, Nagoya 464-8601, Japan
[b]PRESTO, Japan Science and Technology Agency (JST), Kawaguchi, Saitama 332-0012, Japan
[c]Laboratory of Molecular Biology, Department of Synthetic Chemistry and Biological Chemistry, Graduate School of Engineering, Kyoto University, Kyoto 615-8510, Japan.

*To whom correspondence should be addressed. Koji Uchida, Ph.D., Laboratory of Food and Biodynamics, Graduate School of Bioagricultural Sciences, Nagoya University, Nagoya 464-8601, Japan. Tel: 81-52-789-4127, Fax: 81-52-789-5296. E-mail: uchidak@agr.nagoya-u.ac.jp

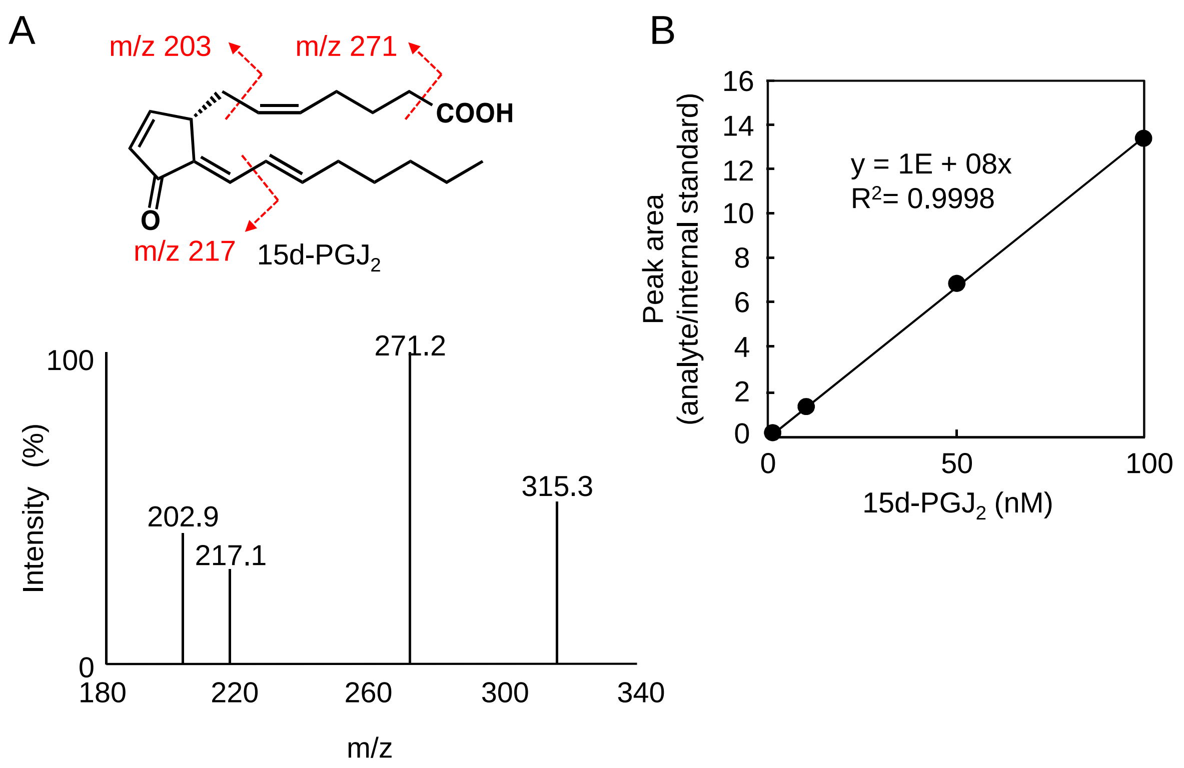

Fig. S1. Quantitative analysis of 15d-PGJ$_2$ using LC-MS/MS.
A, Product ion scan (precursor as *m/z* 315.4) of 15d-PGJ$_2$. B, Calibration curves for 15d-PGJ$_2$.

Supplementary data Fig. S1
Shibata et al.

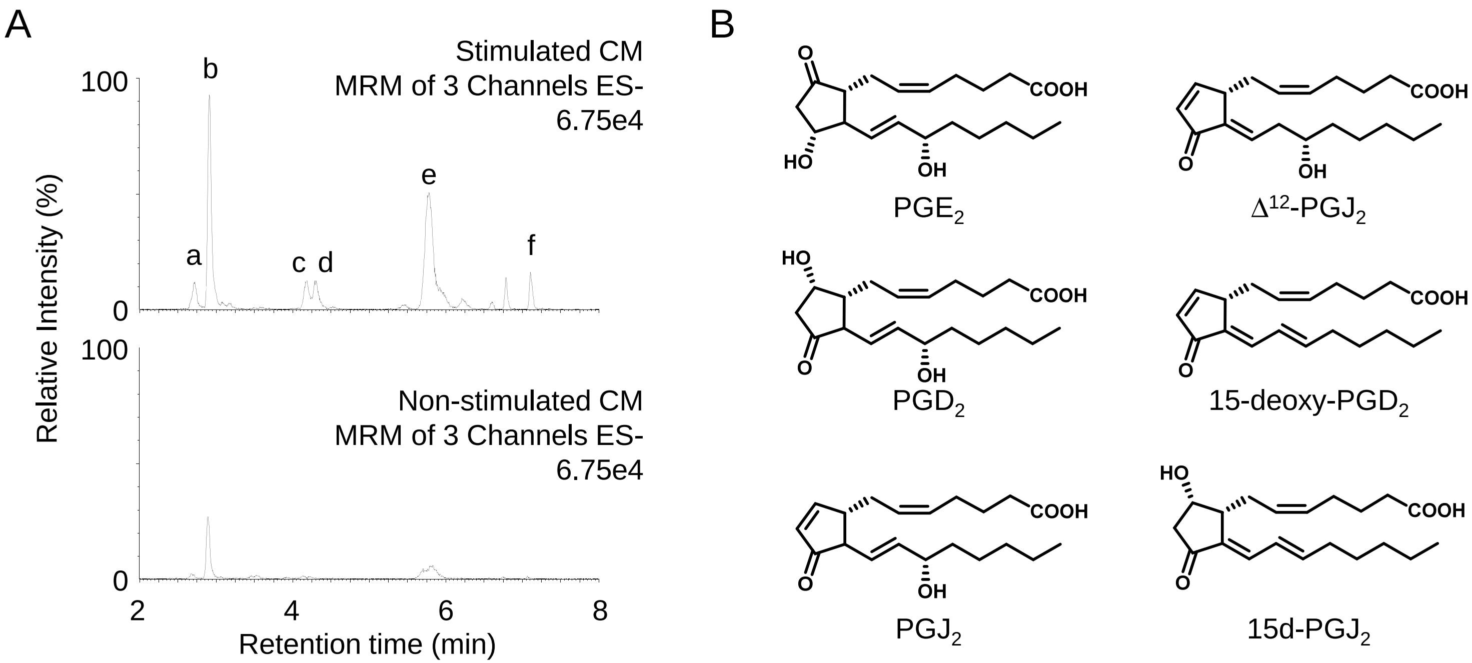

Fig. S2. LC-ESI-MS/MS analysis of the PGs in the conditioned medium from mast cells. A, Total ion current of three mass transitions/channels; 315.2>271.2, 333.2>271.2, 351.2>271.2. *Upper*, conditioned medium (CM) from antibody and antigen-treated cells; *Lower*, CM from non-stimulated cells. a, PGE_2; b, PGD_2; c, PGJ_2; d, Δ^{12}-PGJ_2; e, 15-deoxy-PGD_2; f, 15d-PGJ_2. B, Chemical structures of PGs.

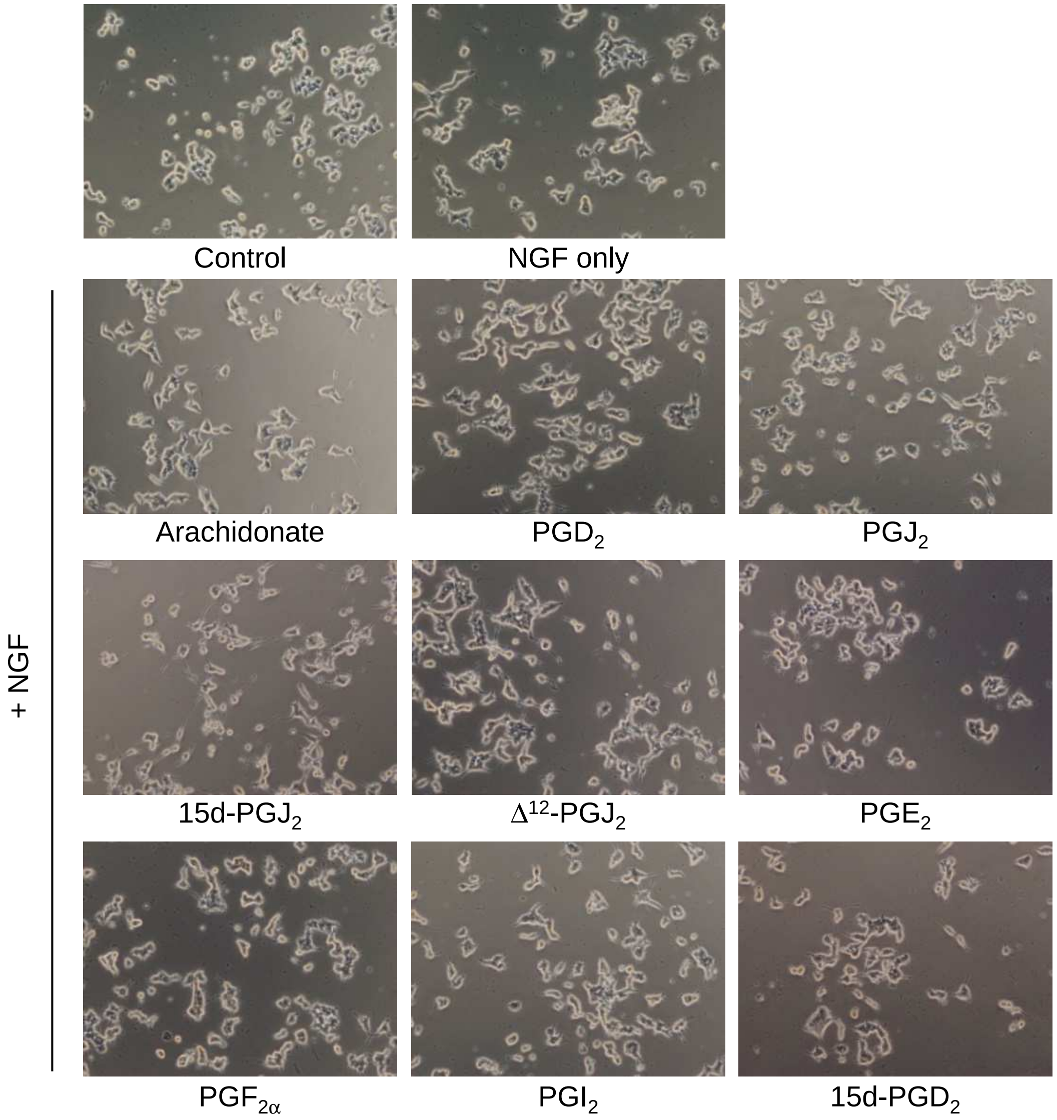

Fig. S3. Representative images of PC12 cells treated with 1 μM PGs in the presence of NGF (1.5 ng/ml) for 72 h.

Supplementary data Fig. S3
Shibata et al.

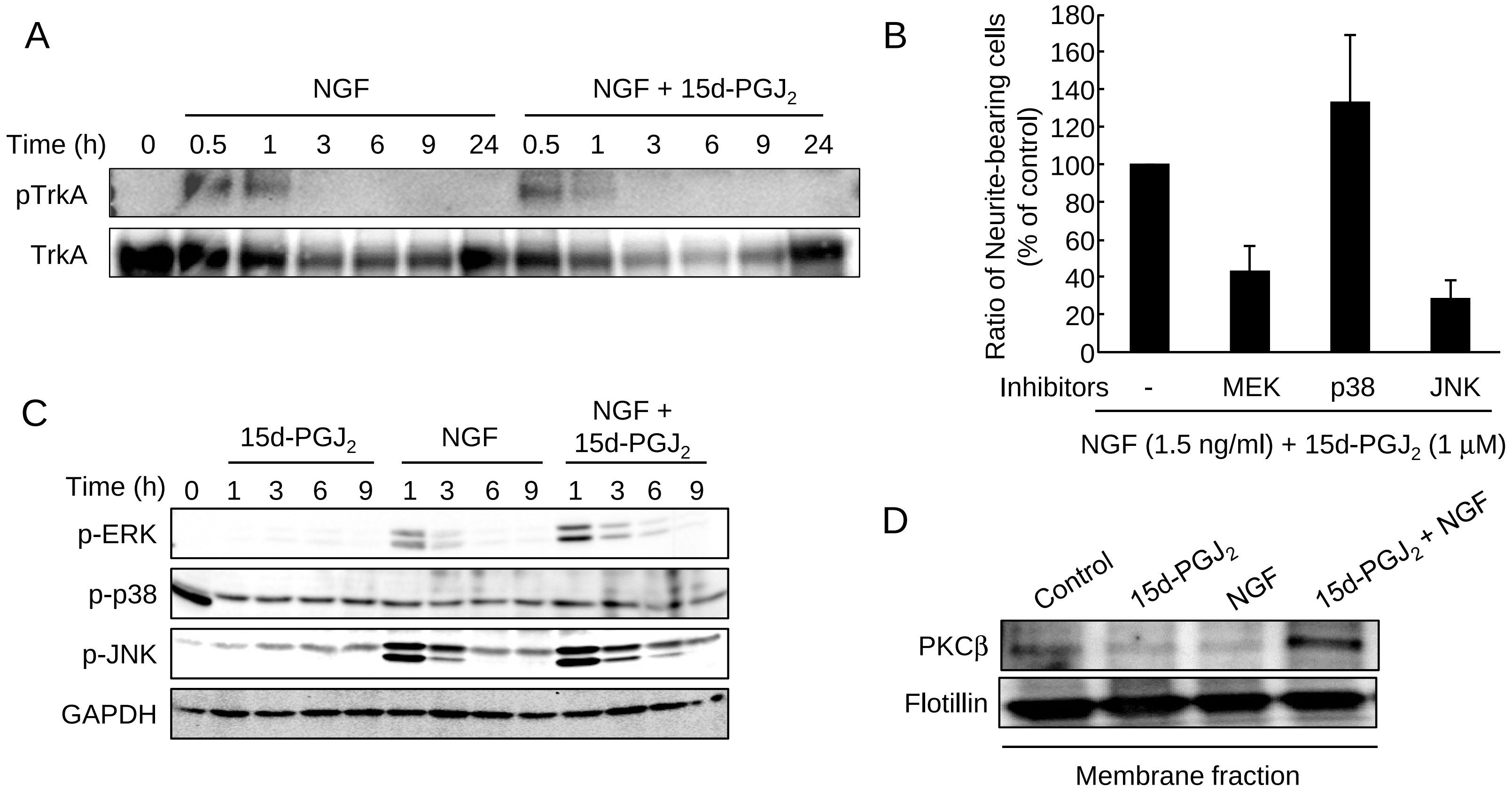

Fig. S4. Involvement of downstream signaling of TrkA-NGF.

A, Immunoblot analysis of phosphorylated TrkA. PC12 cells were treated with 15d-PGJ_2 (1 μM) in the presence of NGF (1.5 ng/ml) for indicated times (0-24 h). B, Immunoblot analysis of activated MAPKs. The cells were treated with 15d-PGJ_2 (1 μM) and/or NGF (1 ng/ml) for indicated times (0-9h). C, Effect of MAPK inhibitors on 15d-PGJ_2/NGF-induced neuritogenesis. The cells were preincubated with each inhibitors (MEK inhibitor PD98059, 20 μM; p38 inhibitor SB203580, 25 μM; JNK inhibitor SP600125 10 μM) for 30 min, and then treated with 15d-PGJ_2 (1 μM) and NGF (1 ng/ml) for 72h. D, Membrane translocation of PKCβ. The cells were treated with 15d-PGJ_2 (1 μM) and NGF (1.5 ng/ml) for 30 min. After treatment, membrane fractions were analyzed by immune blot with anti-PKCβ (upper) and flotillin (lower) antibodies.

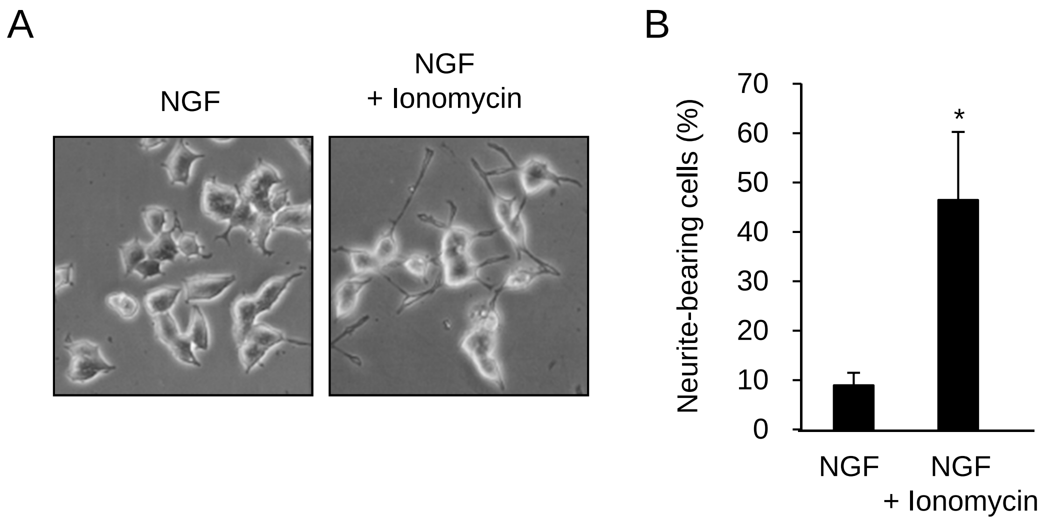

Fig. S5. Effect of calcium ionophore on NGF-induced neuritogenesis.
PC12 cells were treated with NGF (1.5 ng/ml) or NGF together with Ionomycin (1 μM) for 72 h. A, Representative images. B, The results shown are means ±SD of three independent experiments. *$p<0.05$.

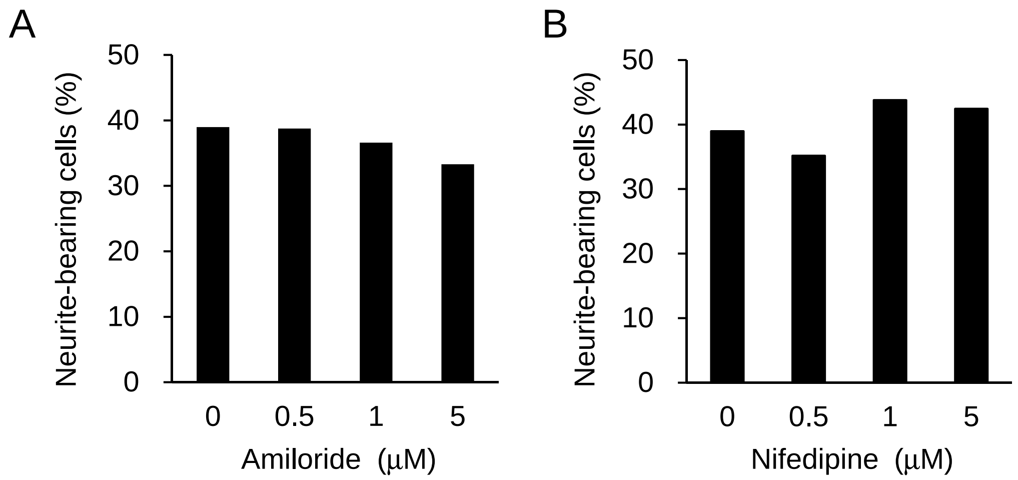

Fig. S6. Effect of channel inhibitors on 15d-PGJ$_2$-enhanced neuritogenesis.

PC12 cells were pretreated with Na^+ channel inhibitor Amiloride (A), or L-type Ca^{2+} channel inhibitor Nifedipine (B) for 30 min and then treated with 1 μM 15d-PGJ$_2$ together with 1.5 ng/ml NGF for 72 h.

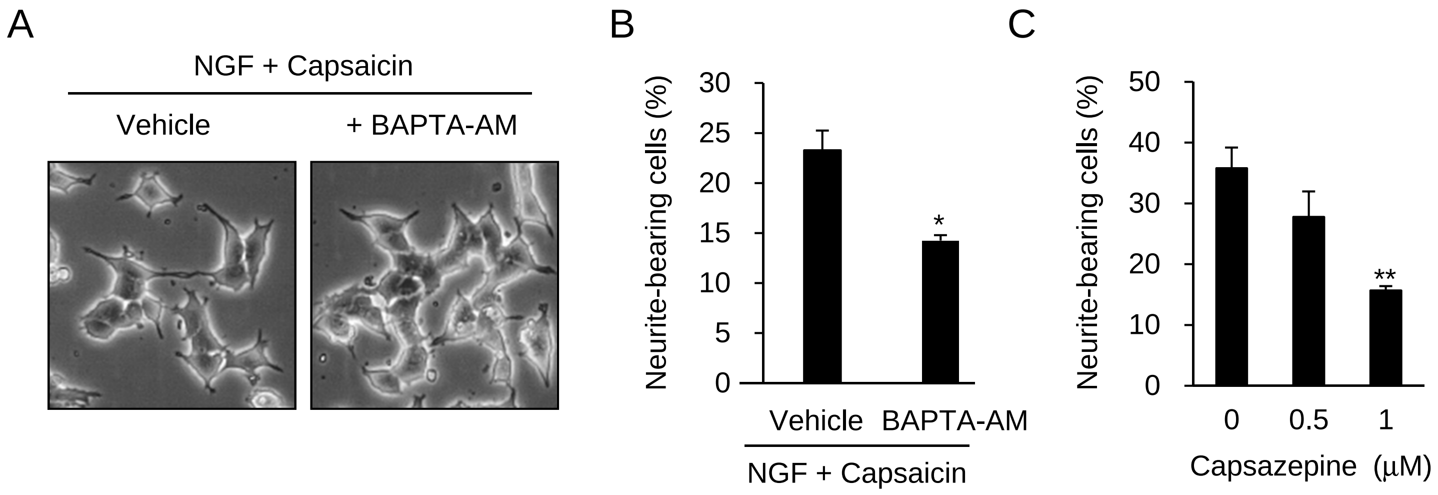

Fig. S7. Involvement of TRPV1 in the neuritogenesis.
A, and B, PC12 cells were pretreated with BAPTA-AM (5 μM) for 30 min and then treated with 1 nM Capsaicin together with 1.5 ng/ml NGF for 72 h. *$p<0.05$. C, DRG cells were pretreated with Capsazepine for 30 min and then treated with 1 μM 15d-PGJ_2 together with 1.5 ng/ml NGF for 72 h. **$p<0.01$.

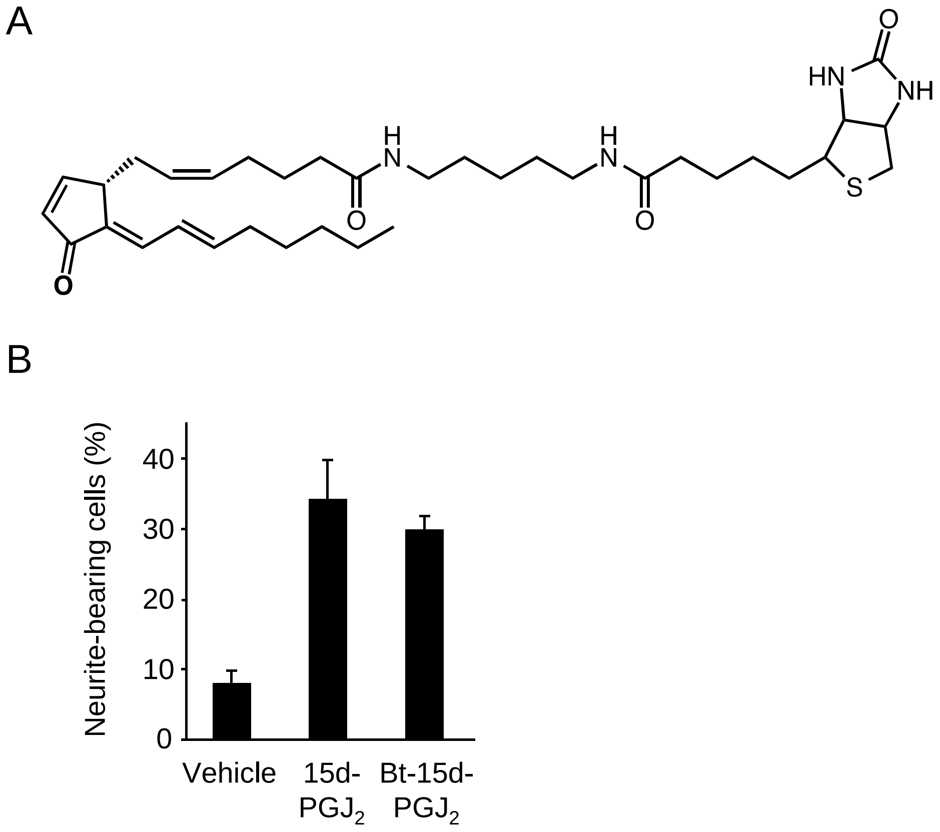

Fig. S8. Effect of 15d-PGJ_2 and its biotinylated derivative on neuritogenesis in PC12 cells.

A, Chemical structure of biotinylated 15d-PGJ_2 (Bt-15d-PGJ_2). B, PC12 cells were treated with 1 μM 15d-PGJ_2 or Bt-15d-PGJ_2 together with 1.5 ng/ml NGF for 72 h.

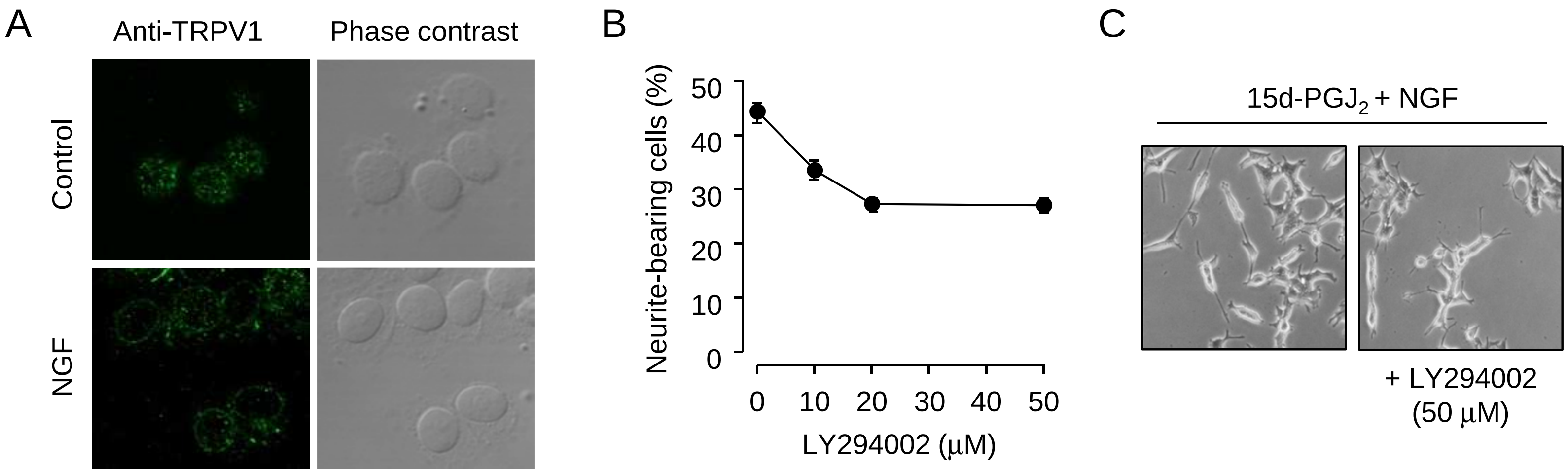

Fig. S9. Involvement of the translocation of TRPV1 into cell surface on 15d-PGJ$_2$-enhanced neuritogenesis.

A, Translocation of TRPV1 into cell surface. The cells were incubated with 1.5 ng/ml NGF for 10 min at 37°C. Alexa488 fluorescence images (TRPV1; geen) are shown in the left column, phase-contrast images are shown in the right column.

B, and C, Effect of PI3K inhibitor LY294003 on the neuritogenesis enhanced by 15d-PGJ$_2$ and NGF. C, Representative images.

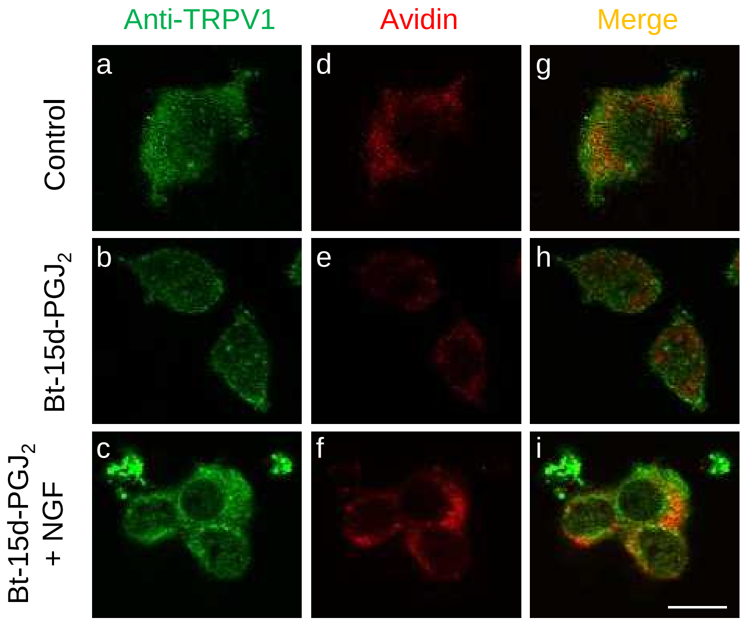

Fig. S10. Immunocytochemical detection of Bt-15d-PGJ$_2$ and TRPV1 in PC12 cells. The cells were incubated with 1 mM Bt-15d-PGJ$_2$ in the absence or presence of NGF (1.5 ng/ml) for 10 min at 37 ^{o}C.Alexa488 fluorescence (TRPV1; green) is shown in the left column of panels a-c, Cy5 (Bt-15d-PGJ$_2$; red) is shown in the center column of panels d–f, and the corresponding merged (superimposed) images are shown in the right column of panels g–i(yellow represents colocalization). Scale bars, 10 mm.

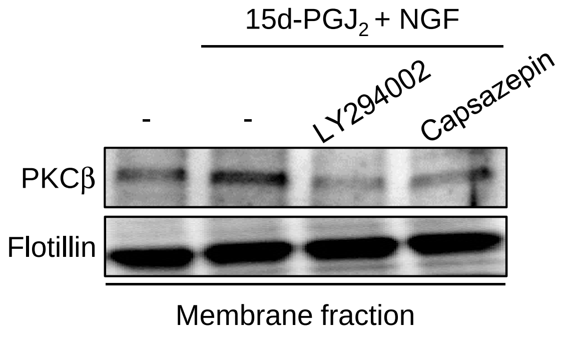

Fig. S11. Effects of PI3K inhibitor LY294002 and TRPV1 antagonist Capsazepine on 15d-PGJ$_2$/NGF-induced membrane translocation of PKC. PC12 cells were pretreated with LY294002 (25 μM) or Capsazepin (5 μM) for 30 min and then treated with 15d-PGJ$_2$ (1 μM) and NGF (1.5 ng/ml) for 30 min. After treatment, membrane fractions were analyzed by immune blot with anti-PKCβ (upper) and flotillin (lower) antibodies.
